# Perceived barriers and enablers to physical activity participation in people with Alopecia Areata: a constructivist grounded theory study

**DOI:** 10.1186/s40359-020-00502-5

**Published:** 2020-12-10

**Authors:** Yamuna Rajoo, J. Wong, I. S. Raj, G. A. Kennedy

**Affiliations:** 1 School of Health and Biomedical Science, RMIT University, Bundoora Campus, Bundoora, Melbourne, VIC 3083 Australia; 2 School of Education, RMIT University, Melbourne, Australia; 3 School of Health and Life Sciences, Federation University, Ballarat, Australia; 4 Institute for Breathing and Sleep, Austin Health, Heidelberg, Australia

**Keywords:** Physical activity, Alopecia areata, Body image, Mental health, Anxiety, Depression, Stress, Constructivist grounded theory, Adjustment and acceptance

## Abstract

**Background:**

Alopecia Areata (AA) is an autoimmune disease that is characterised by hair loss. Individuals diagnosed with it often describe feelings of trauma and social rejection due to cosmetic repercussions and are at high risk of experiencing psychological distress. Physical activity (PA) participation has been associated with better mental health outcomes in diverse populations. A preliminary study of individuals with AA indicated that severe hair loss is associated with symptomatic depression, anxiety and stress, which negatively impacted PA participation. While strategies to increase PA participation in the general population have been established, little is known about PA participation in people with AA. This study aimed to understand barriers and enablers to PA participation in people with AA to inform the development of evidence-based interventions.

**Methods:**

The study used a grounded theory (GT) methodology, relying on an iterative and simultaneous process of data collection, coding, theory development, and data comparisons to explore the perceived barriers and enablers to PA. Data were collected through a focus group (8 participants [33.38 ± 10.81 years]) and individual telephone interviews (8 participants [33.89 ± 11.87 years]). The study was conducted in Melbourne, Australia. Interview data were recorded digitally, transcribed verbatim and analysed. Recruitment continued until theoretical saturation was achieved.

**Results:**

The constructivist grounded theory method used has assisted to develop an explanatory model which is used to explain the themes for barriers and enablers to PA participation. The four phases in the explanatory model are as follows (1) onset of AA; (2) reaction towards the condition; (3) adjustment; and (4) acceptance.

**Conclusion:**

The findings highlighted perceived barriers and enablers to PA participation in people with AA. Future interventions could consider addressing these barriers specifically to maximise effectiveness and to improve mental health status based on the phases of the explanatory model.

## Introduction

Alopecia Areata (AA) is an autoimmune-mediated disease that causes non-scarring hair loss with a prevalence of 0.1–0.2% and lifetime incidence of 2.1% [1]. The scalp is most commonly involved in clinically treated AA, but any hair-bearing surface of the body may be affected [2]. Hair loss ranges from patchy hair loss (AA), hair loss involving the entire scalp (alopecia totalis or AT) or complete body hair loss (alopecia universalis or AU) [3]. There are numerous treatment options available, but the effects of these on hair loss are typically short-term, which, in turn, negatively affects the emotions and mental health of people with AA [4, 5]. People with AA often report being harassed, stared at, or assumed to be cancer patients due to their hair loss [6]. Wigs are a common method used to conceal hair loss which helps minimise the psychological consequences of AA. [7] The use of wigs offers an immediate and efficient cosmetic result and represents a relevant part of the treatments when there is no efficient treatment available. They may reduce potential experiences of social stigmatisation and improve quality of life [8]. However, the psychological effects of such practices are complex; concealing hair loss can increase self‐confidence, but concerns about wigs being noticed or coming off can simultaneously increase anxiety and stress, create feelings of inauthenticity and affect interactions with others, leading to avoidance of social activities particularly those involving physical activity [8, 9].

There is evidence that people with mental health conditions are less active than the general population [10]. The reason for less PA participation includes the mental health condition, specific attitudes towards PA, preferences, fears of discrimination and physical safety concerns [11, 12]. A previous study in people with AA indicated that 81.9% of the participants did not meet PA guidelines [4]. Furthermore, those with more than 50% scalp hair loss and did not meet PA guidelines were significantly more likely to experience symptoms of severe depression, moderate anxiety, and mild stress than those who met guidelines [4].

Although the importance of psychological factors has begun to be recognised in AA as studies have provided insights about psychological well-being and other aspects of the condition, there are important gaps in our understanding about how this condition affects PA participation. Mental well-being was strongly correlated with PA adherence in different populations with mental health conditions [13]. Preferences for the structure of PA (e.g., type of activity, setting, intensity, etc.) vary widely across populations [14]. PA interventions targeting individuals with AA are optimised if barriers and enablers are better understood. Hence, qualitative research is an ideal method for studying the lived experience of people with AA. This study aimed to identify the perceived barriers and enablers to PA participation in individuals with AA by using grounded theory to inform subsequent PA intervention designs.

## Methods

### Sampling method and recruitment

Sampling in grounded theory is sequential, hence selective sampling addressing inclusion criteria was done before the study and theoretical sampling was done when concepts began to emerge [15]. Two data collection methods were used; focus groups and semi-structured individual telephone interviews were conducted in Melbourne, Australia from August to November 2018. Individuals with AA were recruited through advertisements on the websites of the Australia Alopecia Areata Foundation (AAAF) and its social media page (https://www.facebook.com/aaafonline/). The inclusion criteria were that individuals were 18 to 65 years old and had been diagnosed with AA by a clinician/dermatologist. They were excluded if they had serious active or uncontrolled diseases that required medical treatment (e.g., chronic obstructive pulmonary disease (COPD), cardiovascular diseases (CHD)), as these may have limited current PA participation rather than barriers arising from AA.

### Focus group and semi-structured telephone interviews

The use of the focus group method enabled, via group interaction, exploration and better refinement of the opinion of each participant and allowed them to speak freely about factors that enabled and limited their PA participation. Participants were contacted through phone calls two weeks before the focus group and were subjected to the screening questions, which informed the researchers if the participants were eligible to participate in this study. If they were eligible and wished to participate in the focus group, an invitation and Participant Information Sheet (PIS) were emailed to them. The researchers (YR and JW) who conducted the individual and focus group interviews received specific training on qualitative research before the study. Participants who were unable to attend the focus group and expressed discomfort about being in group settings were interviewed via telephone. Interview times were arranged to suit the participants’ schedules. Verbal consent was obtained via telephone and the process was audio recorded. The interviews were recorded using a digital voice recorder (Olympus WS-852). Interview guides were used to maintain direction in conducting individual and focus group interviews. All data from the interviews and focus groups were transcribed verbatim. No further interviews or focus groups were conducted when data saturation was reached; that is, no further new themes or ideas were emerging.

### Interview guides

Due to the exploratory and flexible nature of the grounded theory method, it is not appropriate to identify a list of predetermined questions that can be systematically applied in interviews. Instead, the interview process should allow for relevant information to emerge from the conversation [16]. Therefore, an open-ended questioning approach was used throughout the interviews and focus groups. Interview guides, listed in Table 1 were adapted from previous studies which investigated common barriers and facilitators to PA in adults with depression [17].

**Table 1:** Interview guides

What is physical activity, and do you think physical activity is important?
How active are you currently? Or have you been active before?
What motivates you to exercise?
What stopped you from engaging in physical activity?
How active would you like to be now?
If you are given opportunities to be active, what would you like to do?
How do you think you think opportunities can be facilitated to get you to be active?

## Data analysis

### Constructivist grounded theory

The constructivist grounded theory methodology was used in this study [15, 18] to understand how notions and thoughts of individuals with AA influence their PA behaviours. It also allowed the construction of an explanatory model of factors predictive of regular physical activity in individuals with AA and guided PA interventions to improve mental health status in these individuals. Grounded theory methods are used to construct theories inductively from the data through an iterative process in which researchers moved back and forth between the data and the developing theory [15]. The study focused on Charmaz’s approach to constructivism GT [15] for data collection and analyses and developing an explanatory model, which puts forward the premise that theory or knowledge cannot take shape in a purely objective manner. Instead, the theory is constructed through the interaction of the researcher and research participant. This process allows researchers to develop a theory that is grounded in the data (i.e., individuals’ lived experiences), rather than from pre-existing ideas. The constructivist grounded theory aims to develop a conceptual understanding of a phenomenon while embracing differences of opinion or perspectives [15]. The qualitative methods and reporting of results adhered to the Consolidated Criteria for Reporting Qualitative Studies (COREQ) [19] guidelines and Standards for Reporting Qualitative Research (SRQR) [20]. The qualitative data were analysed using the NVivo software package. The accuracy of the transcripts was verified by cross-checking against the interview recordings. Pseudonyms were assigned to each participant’s information, so their real names were not disclosed, and their identities were protected.

### Data coding

Coding processes are closely tied to theoretical sampling in grounded theory. Grounded theorists have identified three types of coding: (1) initial (2) focused and (3) axial [15]. Initial coding was done by line-by-line or word-by-word examination of the data for developing provisional concepts. Coding was carried out following procedures outlined by Charmaz [15] and facilitated using the “nodes” function in NVivo. The first stage involved initial coding, whereby codes were closely linked to the data. This process allowed the first author (YR) to develop a broad understanding of the data. As data collection and analyses continued, initial codes were refined. Through this process, these concepts were collapsed into categories. In axial coding, the analysis is specifically focused on an emerging category. Selective coding is the examination of the data for unearthing the core categories and achieving the integration of the explanatory model. Constant comparison methods [21] and memo writing [15] were used at all stages of analyses. Sampling and analyses continued until theoretical saturation was achieved (i.e., when data did not reveal any further theoretical insights) and a final explanatory model was developed. All analyses were primarily conducted by the author (YR). To ensure rigour, analyses were continuously and discursively checked by the other authors (GK, JW and ISR), and agreement over themes was reached by consensus.

## Results

A total of 16 individuals with AA participated in this study through a focus group (8 participants [33.38 ± 10.81 years]) and individual telephone interviews (8 participants [33.89 ± 11.87 years]) as shown in Table 2. The focus group lasted about 90 min while the mean duration for each telephone interview was approximately 11 min.

**Table 2:** Participants’ characteristics

Pseudonym	Age range (years)	Type of Alopecia	Percentage of hair loss in the scalp	Years active with AA	Interview method
Andy	45–59	Areata	Patchy	20	TI
Julia	30–44	Areata	Less than 10%	3	TI
Amanda	18–29	Areata	10 to 25%	3	TI
Daisy	45–59	Areata	10 to 25%	11	FG
Mary	18–29	Areata	10 to 25%	3	FG
Katty	30–44	Universalis	50% and above	19	FG
Betty	18–29	Universalis	50% and above	13.5	FG
Any	18–29	Areata	50% and above	5	FG
Charlie	45–59	Universalis	50% and above	30	FG
Tom	18–29	Areata	50% and above	1	FG
Casey	18–29	Areata	50% and above	16	FG
Margaret	30–44	Universalis	50% and above	5.5	TI
Gloria	30–44	Totalis	50% and above	10	TI
Jane	18–29	Areata	50% and above	2	TI
Andrew	30–44	Areata	50% and above	18	TI
Jay	18–29	Areata	50% and above	20	TI

Tables 3 and 4 show the themes related to barriers and enablers to PA participation respectively. Themes are not intended to be mutually exclusive but cut across and between the different levels of influence.

**Table 3:** Barriers to physical activity participation

Themes	Female	Male
Being stared at	But I do get some funny looks after I go to the swim (Daisy, FG) People should stop caring about how other people look like! (Betty, FG)	I feel like people are looking at me. People look at you like you are cancer patient, so they think like I shouldn't be here (at the gym) (Andrew, TI)
Body Image	I would probably still rather go somewhere further away from home because I know a lot of people in my area, so I wouldn't want someone to see me (Betty, FG) I did stop running because of Alopecia. Your body is not handling if you are losing your hair (Mary, FG) When I had hair before and I was sort of more establish like I did play netball, I did all sort of sports as well and then when it started falling out, I sort of stopped doing all of that (Any, FG) I have been consumed by alopecia. I didn't want to be doing sports, I didn't want anyone to find out, I stopped doing personal training and playing netball and few other things (Betty, FG)	Where I was in a small town, they did not understand what was going on with me, so I shun social activities certainly sports is one of them (Charlie, FG)
Managing the noticeability of the wig	But like playing sports, I probably wouldn't play sport rather than wear my wig and play. and you kind of feel like your hair line isn’t in the right spot. So, it does hold me back from doing sports and things like that (Betty, FG) I would like to do a body combat class. But I would always go to the side of the class because it is my safety corner so that I can make a quick exit without being stared (Katty, FG) Many years ago, I used to ride a bike and I had never ridden it since I had Alopecia because it's too uncomfortable with putting a helmet on a bald head and then when you wear a wig and you have to put the helmet on top of it and you don’t have circulation there. (Daisy, FG)	
Restricted dress code	I love doing gymnastics and being on stage, and gymnastics is all about being beautiful and having hair, I can’t do that because I have no hair (Katty, FG) You can’t really many sports with the hat on because it is the uniform policies. I was invited to play mixed netball, I didnt want to play because I couldnt wear a hat because team sports and it is part of uniform regulation (Jane, TI)	
Psychosocial-Being self-conscious and embarrassed	When AA was at worst, I wouldn’t be able to tie my hair up like it was and it made me very self-conscious. So, I wouldn’t go to the gym as often as I would now (Julia, TI) Everything I love to do is too difficult and it’s too embarrassing. I would like to the gym but it’s too embarrassing (Daisy, FG) I used to run in the treadmill, but it just got too embarrassing and I am too conscious of people who is walking around me because my hair would look bad (Gloria, TI)	I guess AA makes you self-conscious in social setting. So, I find it hard to have conversation to new people. that fear of them asking what you look like things like that and the feeling you must explain them is quite difficult (Andrew, TI)
Extreme temperature	My physical activity level when is lesser now compared to before AA because wearing hat and wigs can make you very very hot or they can fall off (Gloria, TI) Last year I didn’t go outside during the weekends for three months because of the wind. That’s why I didn’t play sport (Casey, FG) Even things like going out for a walk in the day, like if its windy day or something, I am scared (Betty, FG)	When I ride bike, I used to wear a sloppy hat and then my helmet and they get all sweaty and horrible (Charlie, FG)

**Table 4:** Enablers to physical activity participation

Themes	Female	Male
Psychosocial—Acceptance of the condition	I had accepted the condition. That helps mentally and I am going really well with it, but when it was at worst and it has been up and down and it’s hard. It’s hard to cope with it, but now I have it for 3 years and I am sort of used to it now (Amanda, TI) I have to go through 16 years of bad and negative mental processess to overcome this condition (Betty, FG) It is sort of also your internal battle with Alopecia. That’s where you must accept yourself and the condition. You can go around and swim bald, but do you want to swim bald? No. So, that’s the inner battle that you must go through yourself and when that time comes if it comes and I will do it (Katty, FG) I will put the hat on and try to not be seen and it shouldn’t' t be that way. It should be going out there and this is me. I am quite there (Katty, FG) Now I am comfortable enough to go out and hang around without a wig even though it had taken a while to be comfortable. I need to do PA to feel better, so I have reached that point where I am not bothered by the environment and what people are thinking (Jane, TI) I had accepted that condition that helps and mentally and I am going well with it, but when it was at worst and it has been up and down, and it is hard. I am quite active now (Amanda, TI) I came to terms with that a little bit better within myself, the motivation just came back, so I do all of those things now (Betty, FG)	The psychological site of it is huge. It took me 20 years to get my head around. It took me a long time to where I am now. (Charlie, FG)
Psychosocial- Social Media and support group	I’ve met my psychologist on that support group, and I have met few other people through that and then that has helped me just to get out there and be a bit more social. That’s a start and then that can transfer to PA (Casey, FG) I wouldnt quit exercise because that is what i something I always do and my family is very supportive of that (Jane, TI)	I have got my wife. She is very fit. She motivates me more than self-motivation (Andrew, TI) I think easier to participate in a group where you are with peers of similar condition. If I were to be in group exercise and all the people have AA, I think it’s good, so more likely you will feel more comfortable being around that sort of people (Andy, TI)
Building resilience from young age	Having that mental resilient despite the visual difference from young is important and the impact AA on the PA participation later in life may be wont be so profound as it was for me which I am trying to make up from my 20 s because I dont have that resilience. So start it from young if they have it from young (Betty, FG)	
Self-Motivation	I am very susceptible to be very down and I have been listening to a podcast and its empowering. It makes me less worry about what people think. I think it is motivating. It works for me (Casey, FG)	
Degree of hair loss	I do not have barriers now because my hair loss isn’t that much, but I think to cope with it pretty well because I tend to change my hairstyle and stuff like that to cover the patches. So I hide it (Amanda, TI)	

FG—Focus group

TI—Telephone interview

**Fig. 1:**
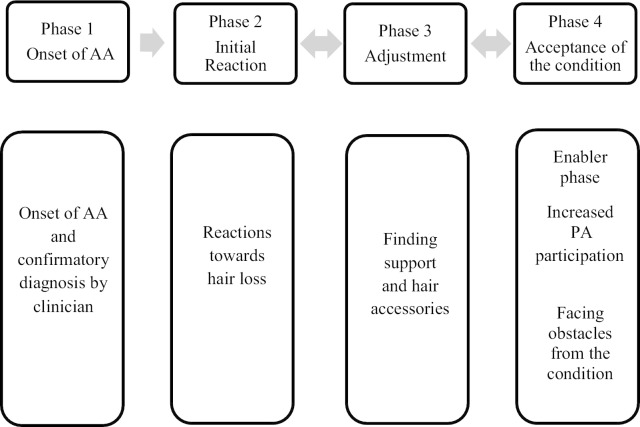
Explanatory model showing the phases of developing acceptance which enables an increase in PA participation

We refined grounded theory and developed an explanatory model of acceptance to increase PA participation by addressing the barriers and enablers in individuals with AA. Findings revealed that individuals who have been diagnosed with AA undergo four phases of changes that start with an onset of AA and ends with behaviours and emotions indicating acceptance towards the condition as shown in Fig. 1. Phase 1 involves symptom recognition, which mainly involves hair loss. This leads participants to visit a clinician or dermatologist to confirm the diagnosis of the condition. Participants’ initial reactions to their symptoms of the condition are detailed in Phase 2. Participants discussed their initial reactions; and their perspective towards PA participation. After experiencing initial reactions, participants undergo a period of adjustment as shown in Phase 3.

While some of these individuals experienced gradual losses from AA to AU or AT, the adjustment process can be prolonged by the new losses and this may cause individuals to be stuck in the adjustment phase where they experience cycles of hope and hopelessness. This phase differs according to individuals whereby they either exhibit negative or positive adjustment. Some participants who are in the adjustment phase wear a wig or hair concealment and still engage in PA, however, the engagement in PA participation is limited. For participants who experienced a longer period of adjustment, finding support helped them in moving towards accepting the condition. In this phase, there are three types of adjustments involved; physical, environmental and psychosocial. These are observed as barriers to PA with a psychosocial adjustment being both an enabler and barrier to PA. Some had accepted their condition with an aid of wig, and some had come out to be bald in Phase 4. Phase 4 was the most important phase whereby participants exhibited higher PA participation, which is seen as an enabling phase.

It is important to note that while this explanatory model presents a path towards accepting the condition to increase PA participation, it is embedded with challenges throughout the journey and varies according to individuals and the duration of living with AA since the first onset. These phases are not linear, and some of the individuals may not experience any of them. Yet and still, others might only undergo one/two or three phases rather than all four. The phases are described in further detail below.

### Definitions of physical activity

Participants were asked to give a personal definition of PA, which encouraged them to reflect on their understanding and experiences. Most of the participants broadly defined PA as any lifestyle activity which involved energy expenditure such as going to the gym, walking, group activities or even playing squash.

#### Phase 1: onset of AA

Typically, the process began after the onset of AA. Often the diagnosis was confirmed by a dermatologist or clinician. In this study, the duration that participants were living with AA typically ranged from one to thirty years and the time of diagnosis varied according to the first onset of AA. Some experienced relapse from AA to AU and AT and back to AA. Hair regrowth varied according to individuals. Once they had been diagnosed with AA, it provided a natural transition to Phase 2, which is the initial reaction towards symptoms of AA.

#### Phase 2: initial reaction

During this phase, nearly half of the participants discussed their initial reactions to the condition. The initial reactions involved physical, psychological and emotional changes in response to the symptoms of the condition. Physical change (hair loss) was viewed as an undesirable attribute with participants reporting experiences of stigmatisation including insensitive encounters, be stared at, and comments about hair loss as a sign of cancer. Suffering from AA may create negative feelings and hence they refrained from activities including PA.

#### Phase 3: adjustment

The adjustment phase was the period in which most participants were dealing with several changes upon being diagnosed with AA. Our analysis of the participants’ accounts led us to identify three themes of barriers to PA; physical, environmental and psychosocial. The theme psychosocial was at times both a barrier and an enabler.

### Physical: body image

Physical adjustment involves adjusting to disfigurement and processes involved in dealing with being visibly different. It mainly involves wearing a wig to conceal hair loss and managing the noticeability of it. Female participants who wore a wig to conceal hair loss expressed their concern with managing the noticeability of the wig. Wearing a wig also led to reduced PA due to concerns about having to take off the wig. Participants described concealing their baldness or patchy hair loss from close acquaintances and feeling unable to be bald at home because of fears that someone might find their appearance disturbing or unattractive. Consequently, wigs, hats, caps and make-up can produce feelings of inauthenticity, shame and anxiety. PA avoidance in these participants is mainly due to the discomfort of disclosing the condition. These behaviours included avoiding social encounters in PA settings by working out at home, somewhere further away from their home or even making a quick exit from the gym. Hair loss or wearing hair accessories such as wigs, hats and caps during PA had caused several participants to avoid PA due to strict uniform or sports policies practised, particularly in team sports such as netball and individual sports such as gymnastics.

### Environmental

Extreme weather conditions such as humidity, rain and wind may also profoundly affect PA participation. Many participants expressed discomfort wearing hair accessories. They indicated wearing a wig during hot days caused overheating while windy weather might create worry of the wig falling off. Similarly, water sports were often avoided due to the need to take the wig or any hair accessories off. Responses identified concerns around the inconvenience of wearing wigs during PA participation.

### Psychosocial

Visible disfigurements can be associated with extensive psychosocial difficulties. A significant proportion of participants experienced psychosocial distress with their visible difference. People struggled with, or avoided, activities ranging from sport and exercise, to shopping, socialising and simply leaving the house because of anxiety that their hair loss would be noticed or exposed. Participants reported feeling more self-conscious about having to wear a wig and being bald. Self-consciousness and embarrassment appeared to arise because of concerns about others finding out they were wearing a wig or concerns about how they look in PA settings. Hair loss had caused emotional devastation for most of the participants, which led to poorer mental health.

### Presence of social support

The presence of social support (family members or friends) or even another individual with AA contributed to PA participation. Relying on social support was mentioned as relevant to deciding to start and continue an activity, especially PA. Social support from family, friends, social media, AAAF support groups across Australia and individuals with similar condition facilitated the development of acceptance towards living with this condition. Online forums, the AAAF website and support groups were frequently mentioned as a crucial source on the journey to accepting the condition. Another coping mechanism includes listening to podcasts as it is empowering and motivates these individuals. PA levels in these individuals often depend on the degree of hair loss.

Participants reported high levels of PA when hair loss was minimal and when they found ways to fix the loss. Other participants adopted a slower pace when engaging in PA allowing them to continue their activities while managing their symptoms*.* When asked about what opportunities can be facilitated to get them to be active, many agreed that group exercise among AA individuals would help them to be more active. Participants also preferred to do exercise with people at their level of ability, and with someone, they knew. An individual also suggested that providing an individualised home-based program as a complement to the group program was an important option for individuals who were uncomfortable in the group setting or who were in rural locations. The duration spent in this phase differed according to individuals. Some stayed in this phase, some took longer to move to the next phase, while others found ways or optimism to move as they stated that, “it’s a journey”. The transition from adjustment to acceptance phase takes a very long time, though it varies according to individuals and is often accompanied by cycles of hope and hopelessness.

#### Phase 4: Acceptance

In this phase, acceptance encompassed participants actively engaging in PA and facing the challenge of their diagnosis. This phase was observed as the enabler phase. Acceptance of the condition plays a major role in PA participation, whereby individuals who accepted the condition were more comfortable in PA settings and had high adherence to PA. A shift was seen from having to adjust to living with a diagnosis they did not ask for and often were devastated to have, to beginning to modify thoughts about their life and future. One participant suggested that building resilience at an early age would help them to accept the condition. The duration of time before accepting the condition varied according to individuals, one participant also explained that it took a long time to accept the condition*.* Participants discussed engaging more in PA when they have accepted the condition and come in terms with it.

## Discussion

This is the first study that examined the perceived barriers and enablers to PA participation in individuals with AA using the constructivist grounded theory aided by an explanatory model. The explanatory model constructed highlights the important role of acceptance in increasing PA participation among individuals with AA. Participants in this study were scattered across the various phases as depicted in Fig. 1, with most of them in the adjustment phase and finding optimism for transitioning to the acceptance phase.

The explanatory model has four phases. The first phase involves the diagnosis of AA which always preceded and provided a natural transition to the second phase. The second phase describes the initial reaction to the symptoms of the condition. Once a diagnosis is made, an individual might experience a range of distress such as depression, anxiety and stress [22]. Anxieties in these individuals often result from describing baldness to the people around them and fears if their appearance is disturbing and/or unattractive to other people [9]. Several barriers to PA were related to psychological distress, including being shy, embarrassed and self-conscious. These identified barriers are consistent with previous research with individuals with mental health conditions [23]. Highly distressed participants may face greater barriers to PA or are less able to problem-solve such barriers, and exercise counselling could be an effective means to facilitate PA adoption in this group [24].

Phase 3 describes the process of adjustment in which participants discussed struggling with physical, environmental and psychosocial changes. As they are unlikely to adapt to the phase preceding acceptance and this phase can last a long time, this stage probably has a negative impact on mental health status. Physically active participants adapted the three aspects of body image coping factors; avoidance, appearance fixing and acceptance [25] to engage in PA or other activities in daily life. Such strategies are less likely to threaten or affect an individual's self-concept and body image. This is seen as the main enabler for PA participation in this study. Appearance fixing concerns efforts to change one's appearance by concealing or correcting a physical feature perceived as flawed. The effort put into concealing hair loss and managing the noticeability of wigs was itself a burden for them. Wearing a wig had a positive impact on PA participation with negative experiences related to managing the wigs. Accessories such as caps, beanies and scarves may also protect the scalp from extreme weather conditions and may help conceal hair loss for an individual with AA which also assisted them to engage in PA, but the psychological effects of such practices are complex. Masking of hair loss can increase self‐confidence, but concerns about wigs being noticed or coming off can simultaneously increase anxiety, create feelings of inauthenticity and affect interactions with others, leading to avoidance of PA. For participants who experienced a positive period of adjustment, finding support aided them in moving toward acceptance. The influence of social support from family, friends, individuals with similar condition and AAAF support groups was brought up as enablers in our study. This underlines the great variability of the role played by them. Friends and family appear as a potential source of increased adherence to PA. This enabler was also observed in another study investigating enablers and barriers to PA involving individuals with chronic low back pain [26].

In the fourth phase, acceptance, participants were ready to face the challenges arising from symptoms of their condition. Acceptance is regarded as a considerable problem in individuals with dermatological complications because when a condition usually occurs unexpectedly, it changes the individual’s outlook on life, causes a revaluation of their life and a change in priorities [27]. This study indicated that accepting the condition plays a crucial role in PA participation. The participants who had accepted the condition continued living a normal life without being affected by AA while others could not come to terms with this new and difficult situation including performing physical activities [27]. For example, a participant explained social encounters at PA settings involving being stared at, subjected to insensitive comments and bullied caused greater self-consciousness and embarrassment. However, explaining that the condition is just hair loss and normalising the encounters can facilitate acceptance and social connection in such settings. Yet, this journey of working towards acceptance was presented as a long struggle by an elderly participant. Another participant indicated the processes involved were often unnecessarily long with a strong influence by positive social factors and by building resilience towards the condition. Most of the participants in this study indicated that acceptance of AA plays a key role in allowing them to adapt to the condition and function normally, and to engage in PA. The journey towards acceptance is influenced by several factors such as personality traits, severity, and duration of the condition which play a vital role to the adaptation and coping process.

This study showed that these participants generally enjoy PA, believe in its benefits and want to be more active regardless of which phase they are in. Commonly endorsed PA are exercises done close to home, in the outdoors and group exercises which are also consistent with previous research with adults with psychosocial distress [28]. The participants agreed that group-based exercise was more attractive and motivating than exercising alone at home. A study indicated that group activities are thought to increase motivation and social interaction and make activities more enjoyable through social support [29]. Motivations to engage in PA often relate to mental and physical health, weight management and fitness, while exercise adherence is more often associated with enjoyment, personal interest and social interaction [30].

A participant suggested that programs for individuals with AA should utilise exercise instructors who can provide a non-judgemental environment, encouragement and professional instruction such as yoga. Maintaining low-stress levels using religion, spirituality or meditation such as yoga may assist with coping was also discussed during the focus group. Relaxation may be able to provide an individual with inner balance, peace and the mental strength to face challenges [31].

Individuals with AA have a diverse range of individual circumstances, and because PA preferences, enablers and barriers are varied over time (e.g. due to changes in condition/hair growth, duration of AA, acceptance, resilience and restrictions due to unpredictable hair loss), broadly targeted and one-size-fits-all interventions may be difficult to justify [32]. A key strength of this study was the application of the constructivist grounded theory, which was used to develop an explanatory model to guide appropriate intervention design. Understanding the PA preferences (type, context and sources of support), enablers and barriers of adults with AA can guide the development of interventions for this group. Strategies to promote physical activity may be more successful if they reflect people's interests [33]. Future intervention design should incorporate acceptance and commitment therapy (ACT) combined with PA. ACT is a psychological therapy that encourages participants to change their relationships with their thoughts and physical sensations through mechanisms of acceptance, mindfulness and value-based action [34]. Incorporating psychological therapy especially ACT [34] together with PA is crucial to maximising the effects of mental health outcome in individuals with AA. The authors acknowledged that this study has several limitations. Firstly, this study has a predominance of female participants and no attempt is made to generalise these results to the general population of people with AA. Secondly, it is recognised that the participants might have psychosocial experiences and coping behaviours different from other individuals with AA. Lastly, the authors acknowledge the limitation in the small number and homogeneity of experiences among these participants.

## Conclusion

The constructivist grounded theory of the current study offers a framework that explains the processes involved to increase PA participation in individuals with AA by addressing the barriers and enablers. Assessment and treatment should focus on the phase preceding acceptance. The provision of effective intervention for individuals with AA requires an understanding of what it is like to live with the condition. Findings from this study can inform intervention and resource development addressing behaviour change related PA for individuals with AA and incorporating it with ACT. People with high levels of distress may require additional support to manage barriers of poor mental health. Given the diverse range of PA attitudes identified, exercise interventions may need to be designed with flexibility for individual attitudes and circumstances.

## Data Availability

Datasets generated and analysed during the current study are not publicly available due to ethics regulations but may be available from the corresponding author upon reasonable request.

## Abbreviations

AA: Alopecia areata
AT: Alopecia totalis
AU: Alopecia universalis
AAAF: Australia Alopecia Areata Foundation
CVD: Cardiovascular Disease
GT: Grounded theory
PA: Physical activity
